# Supplementary material for: Analysis of PPARγ Signaling Activity in Psoriasis
Source: Int J Mol Sci. 2021 Aug 10;22(16):8603. doi: 10.3390/ijms22168603 (PMC8395241; doi:10.3390/ijms22168603)
Supplement: Supplementary file 1 [file ijms-22-08603-s001.zip › Supplemental materials_Analysis of PPARg signaling activity in psoriasis/Pathway models/Models images and html files/PPARG inhibits phenotypes assotiated with psoriasis/245050.html]

monocyte infiltration


# Disease monocyte infiltration

|  |  |
| --- | --- |
| URN | urn:agi-disease:monocyte%20infiltration |
| Total Entities | 0 |
| Connectivity | 1218 |
| Name | monocyte infiltration |

---

|  |  |
| --- | --- |
| Pathway | PPARG inhibits phenotypes assotiated with psoriasis |
|  | efects selected |

---

|  |  |
| --- | --- |
| MedScan ID | 9017768 |
|  | 4999999 |
|  | 4999996 |

---

|  |  |
| --- | --- |
| Alias | monocyte infiltrations |
|  | monoblastic infiltration |
|  | monocytic infiltrations |
|  | monocyte infiltration |
|  | infiltration by monocytes |
|  | monocytic infiltration |
|  | mononuclear cell infiltrations |
|  | monocyte infiltrating |
|  | monoblast infiltration |
|  | peripheral blood mononuclear cell infiltration |
|  | infiltration by mononuclear cells |
|  | mononuclear cell infiltration |
|  | mononuclear cell infiltrating |

---

|  |  |
| --- | --- |
| CellType | monocyte {CellType urn:agi-ncimcelltype:C0026473} |
|  | mononuclear cell {CellType urn:agi-ncimcelltype:C0806987} |
|  | monocyte |
|  | mononuclear cell |

---

|  |  |
| --- | --- |
| CellProcess | cell infiltration {CellProcess urn:agi-cellproc:cell%20infiltration} |

---
